# The role of ultra-processed food consumption in protein-energy wasting and sarcopenia development in patients with chronic kidney diseases

**DOI:** 10.1186/s12882-023-03409-6

**Published:** 2024-01-03

**Authors:** Zainab Shateri, Sevda Eskandarzadeh, Mehran Nouri, Shahrokh Ezzatzadegan Jahromi, Fatemeh Mansouri, Siavash Babajafari

**Affiliations:** 1 Department of Nutrition and Biochemistry, School of Medicine, Ilam University of Medical Sciences, Ilam, Iran; 2 Department of Nutrition, School of Public Health, Iran University of Medical Sciences, Tehran, Iran; 3 Student Research Committee, Shiraz University of Medical Sciences, Shiraz, Iran; 4 Health Policy Research Center, Institute of Health, Shiraz University of Medical Sciences, Shiraz, Iran; 5 Department of Community Nutrition, School of Nutrition and Food Sciences, Shiraz University of Medical Sciences, Shiraz, Iran; 6 Department of Medicine, School of Medicine, Shiraz Nephro-Urology Research Center, Shiraz University of Medical Sciences, Shiraz, Iran; 7 Department of Clinical Nutrition, School of Nutrition and Food Sciences, Shiraz University of Medical Sciences, Shiraz, Iran; 8 Department of Clinical Nutrition, Nutrition Research Center, School of Nutrition and Food Sciences, Shiraz University of Medical Sciences, Shiraz, Iran

**Keywords:** Ultra-processed foods, Protein-energy wasting, Sarcopenia, Chronic kidney disease

## Abstract

**Background:**

The effect of ultra-processed foods (UPFs) on chronic kidney disease (CKD) has been studied in some studies. The present study aimed to investigate the association between UPF consumption and the risk of protein-energy wasting (PEW) and sarcopenia in patients with CKD in the Iranian population.

**Methods:**

The current cross-sectional study included 110 patients with CKD referred to two clinics in Shiraz, Iran. The International Society of Renal Nutrition and Metabolism (ISRNM) criteria and the Asian Working Group for Sarcopenia (AWGS) guideline were considered for the diagnosis of PEW and sarcopenia, respectively. The valid semi-quantitative food frequency questionnaire was used to assess participants' dietary intake. The logistic regression was used to examine the association of UPFs with PEW and sarcopenia.

**Results:**

We observed no significant association between sarcopenia and PEW with UPFs in the crude model. After adjusting for confounders, we observed a significantly higher odds of sarcopenia in the upper versus lower median of UPF intake (odds ratio (OR) = 3.59, 95% confidence interval (CI): 1.02–12.62, P = 0.046).

**Conclusions:**

Our findings suggest a positive relationship between UPF intake and sarcopenia among CKD patients. Therefore, reducing the intake of UPFs may decrease the odds of sarcopenia in patients suffering from CKD.

**Supplementary Information:**

The online version contains supplementary material available at 10.1186/s12882-023-03409-6.

## Introduction

Chronic kidney disease (CKD) is one of the most important chronic non-communicable diseases whose prevalence and incidence are increasing [1]. The global burden of CKD increases mainly due to aging, diabetes, infections, hypertension, environmental toxins, and high body mass index (BMI) [2]. The prevalence of this disease is between 11.7% and 15.1% in the world [2].

One of the crucial complications of CKD is protein-energy wasting (PEW), a condition of metabolic and nutritional disorders in which the body's energy reserves and systemic protein are lost simultaneously [3]. PEW occurs due to anorexia, autoimmune conditions, systemic inflammation, and a hypercatabolic state due to uremia [4]. Sarcopenia is another common complication of CKD, with decreased muscle mass, performance, and muscle strength. This disease is associated with cardiovascular complications and increased patient mortality [5].

Diet plays an essential role in CKD. In CKD patients, phosphorus, sodium, and protein consumption is reduced to delay the onset of uremic toxicity, which may have toxic effects on the kidney, heart, and blood vessels [6]. The nutrition transition is happening rapidly in Iran [7]. This concept shows an increased tendency to consume hydrogenated fats, animal fats, sugar-containing products, packaged snacks, low-fiber foods, artificially sweetened beverages, and processed foods. In fact, the modern diet has replaced the traditional diet [8, 9]. Ultra-processed foods (UPFs) are ready-to-heat or ready-to-eat foods [10]; many of these foods are considered nutritionally poor, containing high levels of free sugars, saturated fat, salt, phosphorus, energy, additives, and low levels of micronutrients, protein, and fiber [11–13].

Many epidemiological studies show that UPFs are harmful to human health [12, 14]. The effect of UPFs on CKD has been studied in some investigated [15, 16]. A cross-sectional study showed that more intake of UPFs was associated to a more prevalence ratio of CKD [17]. In a cohort study, the findings indicated that higher consumption of UPFs was associated with a higher risk of CKD [18]. The pathways by which UPF intake may lead to the onset of CKD are not fully understood. However, an animal study suggests that advanced glycation end products (AGEs) derived from processed foods are an important factor in the pathogenesis of CKD by activating the complement pathway and disrupting intestinal barrier permeability [19].

Although there are studies on the effect of UPFs on the risk of CKD, to our knowledge, no study has been conducted on the association of UPF consumption with two important complications of CKD (PEW and sarcopenia). Therefore, the present study aimed to investigate the association between UPF consumption and the odds of PEW and sarcopenia in patients with CKD in the Iranian population.

## Methods

### Study population

Based on the inclusion and exclusion criteria, the current cross-sectional study included 110 patients with CKD referred to Imam Reza and Motahari clinics in Shiraz (Fars province, Iran). Using statistical software and considering the sample size formula for estimating a proportion, α = 0.05, β = 20%, ratio (*p*) = 0.5, and 13% drop-out, 110 patients were determined as our final sample size. The present study was conducted between January and October 2022.

Our inclusion criteria were as follows: aged > 18 years, estimated glomerular filtration rate (eGFR) < 60 mL/min/173m^2^ by a physician, no cognitive impairments, and willingness to participate in the study.

**Fig. 1:**
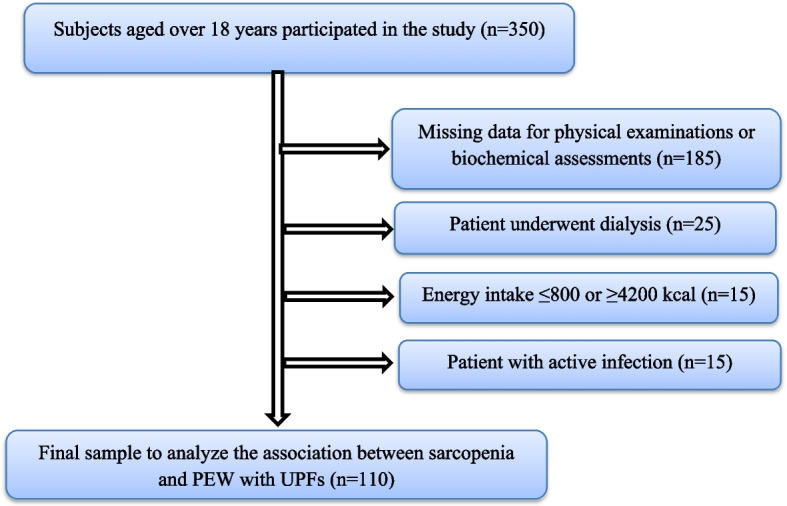
Study flow diagram

Also, our exclusion criteria were as follows: undergoing dialysis, suffering from heart failure and liver cirrhosis, presence of active infection, a daily energy intake of ≤ 800 or ≥ 4200 kcal, and remaining more than 40% of the food frequency questionnaire (FFQ) unanswered (Fig. 1). This study was approved by the Medical Research and Ethics Committee of Shiraz University of Medical Sciences (Code: 28190).

### Data collection

We used bioelectrical impedance analysis (BIA) (IN BODY-S10) to assess muscle mass and fat percentage and the Omron BF511 scale for weight assessment (with minimal clothes, without shoes, and accuracy of 100 gr). A non-stretchable tape (accuracy of 0.5 cm) was used to measure height and mid-arm circumference (MAC). BMI was calculated by dividing body weight by the square of height. We also collected 5 mL of fasting blood samples from all participants to evaluate creatinine, albumin, and other biochemical variables. Moreover, the physical activity level was assessed by a validated international physical activity questionnaire (IPAQ) [20].

### Diagnosis of sarcopenia

The Asian Working Group for Sarcopenia (AWGS) guideline, which considers low muscle mass, low muscle strength, and/or low muscle function as criteria for sarcopenia diagnosis, was used in our study. A dynamometer set measured handgrip strength (HGS) as an indicator of muscle strength. Also, to measure muscle and fat mass, a BIA was used and to assess muscle function, the 5-time chair standing test and the gait speed test were applied (walking speed for 6 m). We considered skeletal muscle mass index (SMI) less than 7 for men and less than 5.7 kg/m^2^ for women as the first criteria for sarcopenia. Secondly, muscle strength less than 28 and 18 for men and women, respectively, and/or weak physical performance (5-time chair standing test more than 12 s or gait speed less than 1 m/s) were considered to approve sarcopenia diagnosis [21].

### Diagnosis of PEW

The International Society of Renal Nutrition and Metabolism (ISRNM) criteria were used for PEW diagnosis. The categories recommended by ISRNM for PEW diagnosis are as follows: low protein and energy intake (dietary protein intake (DPI) < 0.6 g/kg/day and dietary energy intake < 25 kcal/kg/day), low BMI (BMI < 23 kg/m^2^, unintentional weight loss: 5% over 3 months or 10% over 6 months, total body fat < 10%), biochemical indicators (serum albumin < 3.8 g/dL, serum pre-albumin < 30 mg/dL, and serum cholesterol < 100 mg/dL), and muscle mass wasting (decreased muscle mass 5% over 3 months or 10% over 6 months, decreased mid-arm muscle circumference area (a decrease of more than 10% compared to the 50^th^ percentile of the reference population)), and creatinine appearance 24-h urine (lower quartile based on sex)). We excluded patients with less than 500 mL/day of urine to eliminate concerns about urine collection errors. At least three indicators were considered for diagnosing kidney disease-related PEW [22].

### Food grouping and dietary assessment

The valid semi-quantitative FFQ (168 items) was used to assess participants' dietary intake [23]. We also offered participants the household measurement system (like plates, bowls, tablespoons, teaspoons, glasses, and cups) and a validated food album for better estimated the type and size of foods [24]. To measure the amount of food item intake, we converted the recorded values of each food item to grams and multiplied them by daily intake frequency.

The UPF index was calculated by selecting food items that the NOVA system defined as UPFs. UPFs include eight groups (1. non-dairy beverages, 2. cakes and cookies, 3. dairy beverages, 4. fast food and processed meats, 5. oil and sauce, 6. sweets, 7. bread, and 8. etc.) and the items of them are as bellows: processed meats, candies, biscuits, cakes, pastries and sweets, buns, packaged bread, salty snacks, ice cream, industrial fruit drinks, sweetened milk-based beverages, margarine, fries, soft drinks, sauces, dressing, and others. The calorie intake of each UPF item was used to calculate the total daily intake of each one. We divided the average daily calorie intake of each item of UPF groups by the total daily energy intake of UPFs, then multiplied by 100 to estimate the portion of each food group to the total UPF intake. Total UPF calories divided by total caloric intake and multiplied by 100 were used as exposure in our study [12, 25]. Nutritionist IV software for Iranians (version 7.0; N-Squared Computing, Salem, OR, USA) was used in the present study.

### Statistical analysis

We used SPSS (version 26) for all the analyses in our study. A p-value less than 0.05 was considered as level of significance. We reported study participants*'* baseline characteristics based on the median of UPFs as a percentage for categorical and mean ± standard deviation (SD) or median (interquartile range (IQR)) for continuous variables. The logistic regression in crude and adjusted models was used to examine the association between PEW and sarcopenia with UPFs. We adjusted age, sex, energy, and physical activity to eliminate their confounding roles in the final model.

## Results

According to Table 1, there were significant differences between sex (*P* = 0.011), muscle mass (*P* = 0.033), fat percentage (*P* = 0.020), estimated glomerular filtration rate (eGFR) (*P* = 0.029), ferritin (*P* = 0.046), parathyroid hormone (PTH) (*P* = 0.003), HCO_3_ (*P* = 0.019), and UPF intake (*P* < 0.001) between the lower and upper median of UPF intake. The baseline characteristics of the study participants are reported in Supplementary Table 1.

**Table 1:** Baseline features of the study population across the median of UPFs

Variables	UPFs (energy %)		
	**M**_**1**_ (***n*** = 54)	**M**_**2**_ (***n*** = 56)	***P***-value
Age (year) ^1^	66.00 (21.00)	64.00 (19.00)	0.163
BMI (kg/m^2^) ^2^	28.25 ± 4.97	27.82 ± 6.28	0.693
Sarcopenia, yes (%) ^3^	5 (9.30)	11 (19.60)	0.101
PEW, yes (%) ^3^	5 (9.30)	11 (19.60)	0.101
Sex, male (%) ^3^	23 (42.60)	37 (66.10)	**0.011**
Muscle weight (kg) ^1^	19.70 (7.00)	22.20 (7.40)	**0.033**
Fat percentage (%) ^2^	29.34 ± 8.69	25.13 ± 10.01	**0.020**
MAC (cm) ^1^	29.00 (6.00)	29.00 (5.00)	0.757
ASM (kg/m^2^) ^2^	8.26 ± 1.39	8.34 ± 1.68	0.763
HGS (kg) ^1^	15.50 (10.00)	18.00 (12.00)	0.091
Walk duration (second) ^1^	7.00 (1.00)	7.00 (2.00)	0.441
Chair sitting (second) ^1^	14.00 (3.30)	13.50 (5.00)	0.521
SGA ^1^	8.50 (4.00)	9.50 (4.00)	0.170
Smoking, yes (%) ^3^	11 (20.40)	12 (21.40)	0.539
Physical activity (%) ^3^			0.124
Low	42 (77.80)	37 (66.10)	
Moderate	12 (22.20)	19 (33.90)	
Marital status (%) ^3^			0.520
Single	3 (5.60)	4 (7.10)	
Married	51 (94.40)	52 (92.90)	
eGFR (mL/min/1.7m^2^) ^2^	29.82 ± 11.26	35.39 ± 14.93	**0.029**
Hemoglobin (gr/dL) ^2^	12.24 ± 2.00	12.83 ± 2.25	0.152
Albumin (gr/dL) ^2^	4.09 ± 0.42	4.08 ± 0.43	0.968
Iron (µg/dL) ^2^	75.05 ± 35.63	70.37 ± 33.96	0.544
Ferritin (ng/mL) ^2^	151.55 ± 139.69	101.79 ± 102.55	**0.046**
TIBC (mcg/dL) ^1^	289.00 (79.00)	331.00 (86.00)	0.097
ALT (IU/L) ^2^	21.57 ± 8.62	23.34 ± 11.87	0.437
AST (IU/L) ^1^	20.00 (9.00)	21.00 (6.00)	0.715
PTH (pg/mL) ^1^	71.20 (59.90)	46.00 (39.10)	**0.003**
Vitamin D_3_ level (ng/mL) ^2^	37.44 ± 15.38	32.25 ± 14.92	0.081
BUN (mg/dL) ^2^	32.77 ± 15.96	31.25 ± 14.84	0.605
Creatinine (mg/dL) ^1^	21.30 (0.99)	1.92 (0.90)	0.669
Urine creatinine (mg/dL) ^2^	868.35 ± 396.07	872.26 ± 394.41	0.959
FBS (mg/dL) ^1^	99.00 (51.00)	96.00 (18.00)	0.736
TG (mg/dL) ^2^	142.78 ± 70.71	158.57 ± 105.15	0.399
Total cholesterol (mg/dL) ^2^	154.04 ± 39.53	149.77 ± 38.17	0.566
LDL-C (mg/dL) ^2^	87.95 ± 26.73	80.45 ± 26.51	0.190
HDL-C (mg/dL) ^2^	42.31 ± 9.65	43.13 ± 15.25	0.766
PCO_2_ (mmHg) ^2^	41.02 ± 5.66	47.42 ± 53.94	0.388
PO_2_ (mmHg) ^1^	29.60 (17.80)	34.60 (15.00)	0.097
HCO_3_ (mmol/L) ^2^	23.38 ± 3.59	21.79 ± 3.42	**0.019**
UPFs (kcal) ^1^	118.10 (62.88)	264.39 (134.60)	**<0.001**

*BMI* Body mass index, *PEW* Protein-energy wasting, *MAC* Mid-arm circumference, *ASM* Appendicular skeletal muscle mass, *HGS* Handgrip strength, *SGA* Subjective global assessment, *eGFR* Estimated glomerular filtration rate, *TIBC* Total iron-binding capacity, *ALT* Alanine transaminase, *AST* Aspartate transaminase, *PTH* Parathyroid hormone, *BUN* Blood urea nitrogen, *FBS* Fasting blood sugar, *TG* Triglyceride, *LDL-C* Low-density lipoprotein cholesterol, *HDL-C* High-density lipoprotein cholesterol, *UPFs* Ultra-processed foods, *PCO*_*2*_ Partial pressure of carbon dioxide, *PO*_*2*_ Partial pressure of oxygen

Values are mean ± SD or median (IQR) for continuous and percentage for categorical variables

^1^Using Mann–Whitney U test for abnormal continuous variables. Values are median (IQR)

^2^Using independent samples T-test for normal continuous variables. Values are mean ± SD

^3^Using chi-square test for categorical variables. Values are percentages

**Fig. 2:**
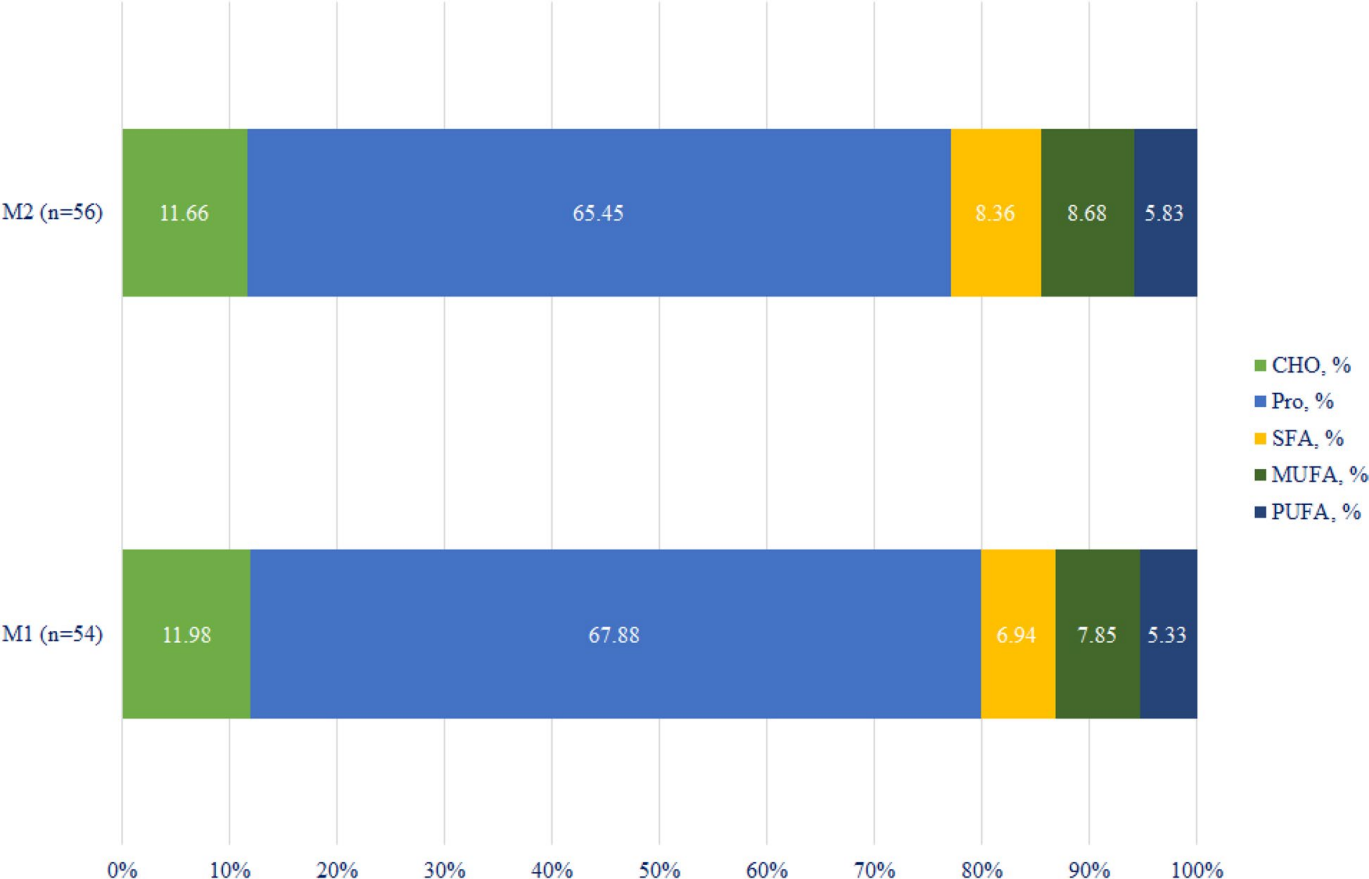
The contribution of macronutrient intake based on UPF median

**Fig. 3:**
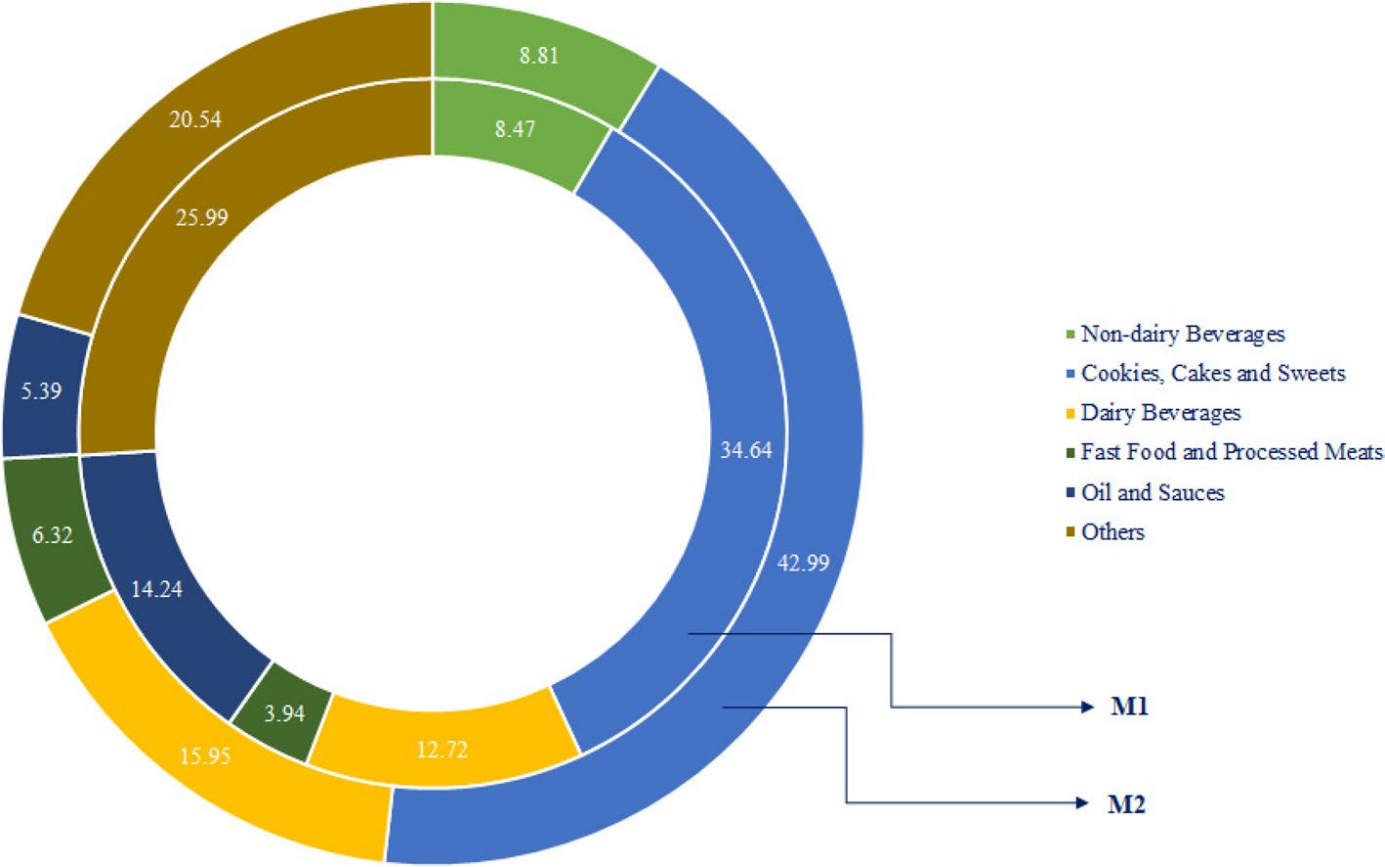
Participants' food group intake across median of UPFs

The intake of macronutrients and UPF subgroups across the median of UPF intake are presented in Figs. 2 and 3. As shown in Fig. 2, the intake of protein, monounsaturated fatty acids (MUFAs), and polyunsaturated fatty acids (PUFAs) did not significantly differ between UPF median (P > 0.05), but there was a significant difference in carbohydrate and saturated fatty acid (SFA) intake (*P* = 0.017 and *P *= 0.014, respectively). The contribution of macro- and micronutrient intake by the median of UPFs is shown in Supplementary Table 2.

In multivariate analysis (Table 2), we observed no significant association between UPFs and sarcopenia and PEW in the crude model. After adjusting for age, sex, smoking, and energy intake, we observed a significantly higher odds of sarcopenia in the upper versus lower median of UPF intake (odds ratio (OR )= 3.59, 95% confidence interval (CI): 1.02–12.62, *P* = 0.046).

**Table 2:** Crude and multivariable-adjusted odds ratios and 95% CIs across the median of energy percent of UPFs

Median of UPFs	Abnormal/normal	Crude Model		Adjusted Model	
		OR	95% CI	OR	95% CI
**Sarcopenia**					
M_1_	5/49	Ref	-	Ref	-
M_2_	11/45	2.39	0.77–7.43	**3.59**	**1.02–12.62**
*P*-value		0.130		**0.046**	
**Protein-energy wasting**					
M_1_	5/49	Ref	-	Ref	-
M_2_	11/45	2.39	0.77–7.43	2.86	0.87–9.40
*P*-value		0.130		0.083	

*UPFs*: Ultra-processed foods

Obtained from logistic regression

These values are odds ratio (95% CIs)

Significant values are shown in bold

Adjusted for age, sex, smoking, and energy intake

## Discussion

Our results showed a direct association between the consumption of UPFs and sarcopenia in adults with CKD after adjusting confounders. According to our results, higher UPF intake leads to 3.59 times increased odds of sarcopenia. However, we did not find any association between the consumption of UPFs and PEW odds.

Evidence suggests that intake of UPFs may cause an increased risk of non-communicable diseases [26]. Based on our knowledge, the current study is the first to assess the association between UPF consumption and sarcopenia and PEW odds in CKD patients.

Some of the characteristics of UPFs, such as affordability, palatability, availability, convenience, and high supply in the market, lead to their overconsumption [27]. UPFs are dense in calories and poor in protein, fiber, vitamins, minerals, and other phytochemicals [28], which are essential nutrients for muscle health [29]. Our study results showed an association between higher consumption of UPFs and sarcopenia in CKD patients. Only one study has investigated the association between these two variables. This cross-sectional study showed that the elderly who consumed UPFs more than 1–2 times a week had a higher chance of developing sarcopenia. Also, their results indicated that the probability of sarcopenia in the elderly increases even with low exposure to UPFs [30].

Wasting of muscle mass, a common criterion of sarcopenia and PEW, is frequently found in CKD patients, especially in those with more advanced stages of the disease. On the other hand, decreased body fat is more common in PEW than in sarcopenia [31]. Etiological factors leading to sarcopenia and PEW associated with CKD that are exacerbated by consumption of UPFs include the following: developed insulin resistance, acidosis, vitamin D deficiency, and disruption of hormones like testosterone, insulin growth factor-1 (IGF-1), and growth hormone (GH) resistance [32, 33]. In addition, the change in the gut microbiome and dysfunction of the gut barrier aggregated by exposure to phthalates, which leaks into food through contact materials or food processing plastics, leads to the development of an inflammatory environment (increased levels of pro-inflammatory cytokines levels such as tumor necrosis factor-α (TNF-α) and interleukin-6 (IL-6)) [34, 35] as a contributor to muscle wasting [36, 37]. One study has reported an association between phthalate exposure and lower grip strength, a marker of muscle health and sarcopenia [38]. Lower fiber intake also leads to the accumulation of protein fermentation metabolites, uremic toxicity [39], and finally, uremic muscle wasting [40].

We did not find a significant association between UPF intake and PEW in patients with CKD. To our knowledge, this is the first study that assessed this association. Mechanisms contributing to PEW are the same as sarcopenia etiology [31], but energy imbalance leading to fat loss could be a more important factor in PEW etiology. Even though, higher PEW was observed in the higher UPF intake group, no significant difference between protein and energy intake was found between the two UPF groups. Furthermore, UPFs contain a high amount of added sugars, trans and SFAs, and additives like emulsifiers, preservatives, stabilizers, and artificial flavors, which enhance lipogenesis (contrary to PEW criteria) and disrupt muscle protein synthesis and ultimately lead to a decline in muscle mass (in line with PEW criteria) [31], resultant of these two may be the reason why we did not observe a significant association between PEW and UPF intake.

We can mention some limitations of the present study. The investigation to the effect of UPF intake on inflammation as a determinant of CKD complications because of the lack of data on inflammatory factors such as nuclear factor-κB (NF-κB), C-reactive protein, and IL-6 were impossible. However, the effects of some confounders were adjusted in the analysis; there may be other unknown confounding factors in this study.

## Conclusions

In conclusion, our findings suggest a positive association between UPF intake and sarcopenia among CKD patients. Therefore, reducing the intake of UPFs may decrease the odds of sarcopenia in patients suffering from CKD. The importance of restricting access to UPFs should be addressed by public policy.

## Availability of data and methods

The datasets used and/or analyzed during the current study are available from the corresponding author on reasonable request.

### Supplementary Information

**Additional file 1:** **Table S1.** Baseline features of the study population. **Table S2.** Macro- and micronutrient intake by the median of UPFs.
