# Supplementary material for: The role of ultra-processed food consumption in protein-energy wasting and sarcopenia development in patients with chronic kidney diseases
Source: BMC Nephrol. 2024 Jan 3;25:4. doi: 10.1186/s12882-023-03409-6 (PMC10763225; doi:10.1186/s12882-023-03409-6)
Supplement: Supplementary file 1 — Additional file 1: Table S1. Baseline features of the study population. Table S2. Macro- and micronutrient intake by the median of UPFs. [file 12882_2023_3409_MOESM1_ESM.docx]

**Supplementary Table 1.** Baseline features of the study population.

| **Variables** | **Median (IQR) or Mean (SD)** |
| --- | --- |
| Age (year) ^1^ | 64.00 (18.00) |
| BMI (kg/m^2^) ^2^ | 28.03 ± 5.65 |
| Sarcopenia, yes (%) ^3^ | 16 (14.50) |
| PEW, yes (%) ^3^ | 16 (14.50) |
| Sex, male (%) ^3^ | 60 (54.50) |
| Muscle weight (kg) ^1^ | 21.15 (8.00) |
| Fat percentage (%) ^2^ | 27.20 ± 9.58 |
| MAC (cm) ^1^ | 29.00 (5.00) |
| ASM (kg/m^2^) ^2^ | 8.30 ± 1.54 |
| HGS (kg) ^1^ | 17.00 (11.00) |
| Walk duration (second) ^1^ | 7.00 (1.00) |
| Chair sitting (second) ^1^ | 14.00 (4.10) |
| SGA ^1^ | 9.00 (4.00) |
| Smoking, yes (%) ^3^ | 23 (20.90) |
| Physical activity (%) ^3^  Low  Moderate | 79 (71.80)  31 (28.20) |
| Marital status (%) ^3^  Single  Married | 7 (6.40)  103 (93.60) |
| **Clinical outcomes** | |
| GFR (mL/min/1.7m^2^) ^2^ | 32.66 ± 13.49 |
| Hemoglobin (gr/dL) ^2^ | 12.54 ± 2.14 |
| Albumin (gr/dL) ^2^ | 4.08 ± 0.42 |
| Iron (µg/dL) ^2^ | 72.60 ± 34.63 |
| Ferritin (ng/ml) ^2^ | 125.44 ± 123.55 |
| TIBC (mcg/dl) ^1^ | 304.50 (82.00) |
| ALT (IU/L) ^2^ | 22.39 ± 10.23 |
| AST (IU/L) ^1^ | 21.00 (9.00) |
| PTH (pg/mL) ^1^ | 63.20 (68.90) |
| Vitamin D_3_ level (ng/mL) ^2^ | 34.74 ± 15.29 |
| BUN (mg/dl) ^2^ | 32.00 ± 15.35 |
| Creatinine (mg/dL) ^1^ | 1.99 (1.03) |
| Urine creatinine (mg/dL) ^2^ | 870.34 ± 393.41 |
| FBS (mg/dL) ^1^ | 98.00 (28.00) |
| TG (mg/dL) ^2^ | 150.76 ± 89.65 |
| Total cholesterol (mg/dL) ^2^ | 151.86 ± 38.72 |
| LDL-C (mg/dL) ^2^ | 84.20 ± 26.74 |
| HDL-C (mg/dL) ^2^ | 42.74 ± 12.79 |
| PCO_2_ (mmHg) ^2^ | 44.28 ± 38.65 |
| PO_2_ (mmHg) ^1^ | 31.00 (17.00) |
| HCO_3_ (mmol/L) ^2^ | 22.57 ± 3.58 |
| **Nutrient and food group intake** | |
| UPFs (kcal) ^1^ | 180.43 (148.55) |
| Energy (kcal/day) ^1^ | 1913.86 (691.33) |
| Protein (gr/day) ^1^ | 52.92 (20.72) |
| Carbohydrate (gr/day) ^1^ | 313.06 (129.97) |
| Fat (gr/day) ^1^ | 48.43 (21.65) |
| Fiber (gr/day) ^2^ | 21.46 ± 5.67 |
| Non-dairy beverages (kcal/day) ^1^ | 7.26 (24.99) |
| Cookie, cakes, and sweets (kcal/day) ^1^ | 64.36 (73.08) |
| Dairy beverages (kcal/day) ^1^ | 25.23 (25.23) |
| Fast foods, processed meats (kcal/day) ^1^ | 3.87 (11.88) |
| Oil and sauces (kcal/day) ^1^ | 12.12 (2.92) |
| Others UPFs (kcal/day) ^1^ | 40.97 (40.97) |

BMI: body mass index, PEW: protein-energy wasting, MAC: mid-arm circumference, ASM: appendicular skeletal muscle mass, HGS: hand grip strength, SGA: subjective global assessment, GFR: glomerular filtration rate, TIBC: total iron-binding capacity, ALT: alanine transaminase, AST: aspartate transaminase, PTH: parathyroid hormone, BUN: blood urea nitrogen, FBS: fasting blood glucose, TG: triglyceride, LDL-C: low-density lipoprotein cholesterol, HDL-C: high-density lipoprotein cholesterol, UPFs: ultra-processed foods.

^1^ Values are median (IQR).

^2^ Values are mean ± SD.

^3^ Values are percentage.

**Supplementary Table 2.** Macro- and micronutrient intake by the median of UPFs.

| **Variables** | **UPFs (energy %)** | | |
| --- | --- | --- | --- |
|  | **M_1_ (n=54)** | **M_2_ (n=56)** | **P-value** |
| Energy (kcal/day) ^1^ | 1774.18 (756.75) | 1993.39 (599.46) | 0.123 |
| Protein (gr/day) ^1^ | 51.11 (27.96) | 56.19 (18.82) | 0.342 |
| Carbohydrate (gr/day) ^1^ | 312.72 (133.95) | 316.62 (128.58) | 0.492 |
| Fat (gr/day) ^1^ | 45.94 (16.87) | 55.12 (22.61) | **0.001** |
| Fiber (gr/day) ^2^ | 20.74 ± 5.50 | 22.15 ± 5.79 | 0.192 |
| SFA (gr/day) ^1^ | 13.09 (9.72) | 16.71 (11.97) | **0.003** |
| MUFA (gr/day) ^1^ | 15.99 (6.77) | 19.18 (9.70) | **0.010** |
| PUFA (gr/day) ^1^ | 10.17 (3.63) | 12.60 (5.53) | **0.003** |
| Sodium (mg/day) ^1^ | 3790.30 (1187.47) | 4019.87 (1209.30) | 0.153 |
| Potassium (mg/day) ^2^ | 2779.92 ±703.69 | 3050.83 ± 765.46 | 0.054 |
| Iron (mg/day) ^1^ | 10.16 (4.85) | 10.80 (4.67) | 0.423 |
| Magnesium (mg/day) ^2^ | 268.20 ±76.92 | 281.10 ±77.94 | 0.384 |
| Zinc (mg/day) ^1^ | 8.96 (4.60) | 9.14 (3.68) | 0.858 |
| Calcium (mg/day) ^1^ | 504.00 (330.37) | 564.69 (158.39) | 0.165 |
| Phosphor (mg/day) ^1^ | 877.40 (423.36) | 1001.85 (272.65) | 0.123 |
| Selenium (mg/day) ^1^ | 35.31 (20.39) | 41.70 (18.19) | **0.036** |
| Vitamin A (RAE/day) ^1^ | 458.95 (244.51) | 560.76 (235.46) | **0.022** |
| Vitamin K (mcg/day) ^1^ | 115.62 (42.52) | 131.52 (57.64) | **0.036** |
| Vitamin E (mg/day) ^1^ | 4.41 (1.97) | 5.45 (2.81) | **0.002** |
| Vitamin C (mg/day) ^1^ | 123.09 (51.69) | 130.16 (51.82) | 0.239 |
| Vitamin B_1_ (mg/day) ^1^ | 0.91 (0.43) | 1.00 (0.42) | 0.209 |
| Vitamin B_2_ (mg/mg/day) ^2^ | 1.07 ± 0.35 | 1.20 ± 0.36 | 0.069 |
| Vitamin B_3_ (mg/day) ^1^ | 34.48 (22.35) | 34.76 (22.42) | 0.566 |
| Vitamin B_5_ (mg/day) ^1^ | 4.71 (1.84) | 4.90 (1.37) | 0.151 |
| Vitamin B_6_ (mg/day) ^2^ | 1.36 ± 0.34 | 1.55 ± 0.40 | **0.009** |
| Vitamin B_9_ (mcg/day) ^2^ | 175.25 ± 53.65 | 208.38 ± 55.66 | **0.002** |
| Vitamin B_12_ (mcg/day) ^1^ | 1.87 (1.21) | 2.12 (1.31) | 0.190 |

M_1_: lower than median intake of UPFs – M_2_: upper than median intake of UPFs.

UPFs: ultra-processed foods, SFA, saturated fatty acid, MUFA: monounsaturated fatty acid, PUFA: polyunsaturated fatty acid.

Values are mean ± SD or median (IQR) for continuous variables.

^1^ Using Mann-Whitney U test for abnormal continuous variables.

^2^ Using independent sample T-test for normal continuous variables.
